# Cellular Auxetic Structures for Mechanical Metamaterials: A Review

**DOI:** 10.3390/s20113132

**Published:** 2020-06-01

**Authors:** Parth Uday Kelkar, Hyun Soo Kim, Kyung-Hoon Cho, Joon Young Kwak, Chong-Yun Kang, Hyun-Cheol Song

**Affiliations:** 1 Center for Electronic Materials, Korea Institute of Science and Technology (KIST), Seoul 02792, Korea; parth.kelkar17@vit.edu (P.U.K.); t19552@kist.re.kr (H.S.K.); jykwak@kist.re.kr (J.Y.K.); cykang@kist.re.kr (C.-Y.K.); 2 Mechanical Engineering, Vishwakarma Institute of Technology, Pune, Maharashtra 411037, India; 3 Quantum Functional Materials Laboratory, Department of Physics, Inha University, Incheon 22212, Korea; 4 School of Materials Science and Engineering, Kumoh National Institute of Technology, Gumi 39177, Korea; khcho@kumoh.ac.kr; 5 KU-KIST Graduate School of Converging Science and Technology, Korea University, Seoul 02841, Korea

**Keywords:** auxetic structure, cellular structure, metamaterial, negative Poisson’s ratio

## Abstract

Recent advances in lithography technology and the spread of 3D printers allow us a facile fabrication of special materials with complicated microstructures. The materials are called “designed materials” or “architectured materials” and provide new opportunities for material development. These materials, which owing to their rationally designed architectures exhibit unusual properties at the micro- and nano-scales, are being widely exploited in the development of modern materials with customized and improved performance. Meta-materials are found to possess superior and unusual properties as regards static modulus (axial stress divided by axial strain), density, energy absorption, smart functionality, and negative Poisson’s ratio (NPR). However, in spite of recent developments, it has only been feasible to fabricate a few such meta-materials and to implement them in practical applications. Against such a backdrop, a broad review of the wide range of cellular auxetic structures for mechanical metamaterials available at our disposal and their potential application areas is important. Classified according to their geometrical configuration, this paper provides a review of cellular auxetic structures. The structures are presented with a view to tap into their potential abilities and leverage multidimensional fabrication advances to facilitate their application in industry. In this review, there is a special emphasis on state-of-the-art applications of these structures in important domains such as sensors and actuators, the medical industry, and defense while touching upon ways to accelerate the material development process.

## 1. Introduction

**Figure 1:**
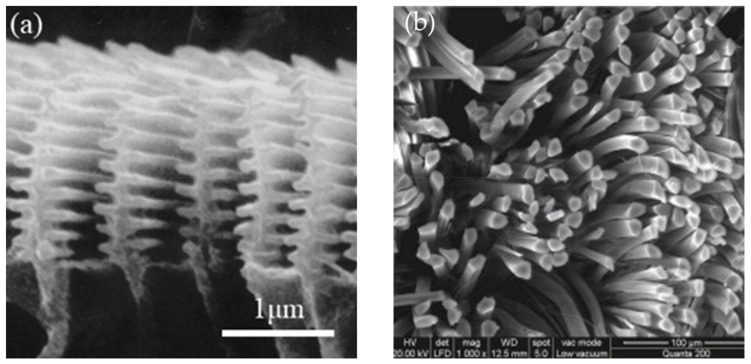
Natural metamaterials. Cross-sectional images of (**a**) the male butterfly *Morpho didius* [5,6], and (**b**) silk fiber [8].

A metamaterial is an artificial material that is designed to obtain particular material properties. As a consequence of their internal geometry and various spatial relations, these materials demonstrate unique and counter-intuitive behavior. The fields of electromagnetic materials (EM) and optics are where the concept of metamaterials was first introduced [1,2,3,4]. Although the metamaterial is a man-made material, it can be found in nature, for example the ‘*morpho*’ butterfly wing [5,6] and silk fiber [7], as shown in Figure 1. Their distinctive microstructure results in their waterproof characteristics or high tensile strength. In this day and age, even if the same material is used, a well-designed microstructure can be the key to unsolved problems, such as the development of self-healing materials, the development of material systems to carry high-density data, and the development of advanced, next-generation sensors.

The most general purpose of metamaterial (or meta structure) is to change the material’s mechanical properties. The four typical material constants of mechanical deformation are the Young’s modulus (*E*), shear modulus (*G*), bulk modulus (*K*), and Poisson’s ratio (*ν*). Unlike the conventional material’s expansion (or contraction) in the directions orthogonal to the applied uniaxial compression (or tension), the behavior of metamaterials is the polar opposite. Materials, which under uni-axial compression contract transversely, and under tension expand transversely, are termed ‘auxetics’, and are characterized by a negative Poisson’s ratio. Greaves et al. (2011) define the Poisson’s ratio as the parameter that “describes the resistance of a material to distort under mechanical load rather than alter in volume” [9]. For isotropic materials, the mechanical constants are related as follows:

(1) $$G=\frac{E}{2\left(1+\nu \right)}$$

(2) $$K=\frac{E}{3\left(1-2\nu \right)}$$

**Figure 2:**
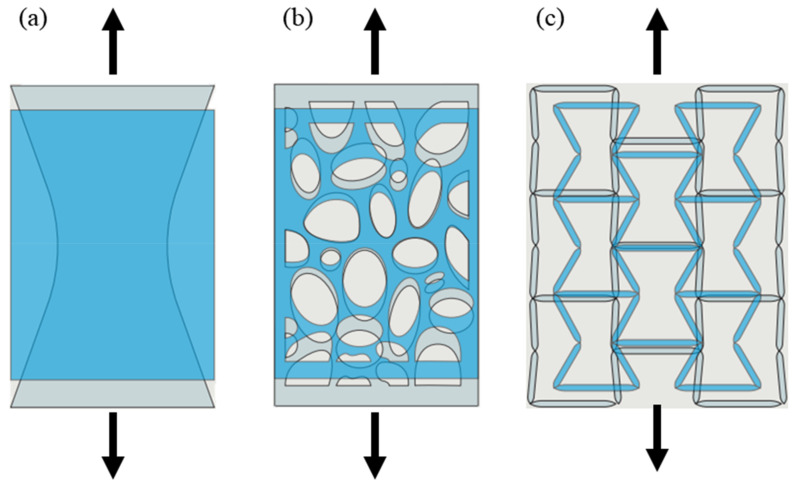
The three types of Poisson’s ratio: (**a**) positive, (**b**) zero, and (**c**) negative [13].

The elastic behavior can be adequately described using two variables. According to Equations (1) and (2), Poisson’s ratio for a stable material is bounded between (–1 and +0.5), due to the pre-requisite that both shear modulus and bulk modulus have positive values [10]. Hence, Poisson’s ratio may have values in the range (–1< *ν* < 0.5) in 3D, and (–1 < *ν* < 1) in 2D. Strain energy density is defined as “the energy that is stored in the material as it is deformed” [11]. Since the strain energy density is positive definite, the Poisson’s ratio for elastic anisotropic materials can have arbitrarily large positive or negative values [12]. Figure 2 shows the three types of Poisson’s ratio. According to the internal geometry of materials, each material has a different Poisson’s ratio. 

**Figure 3:**
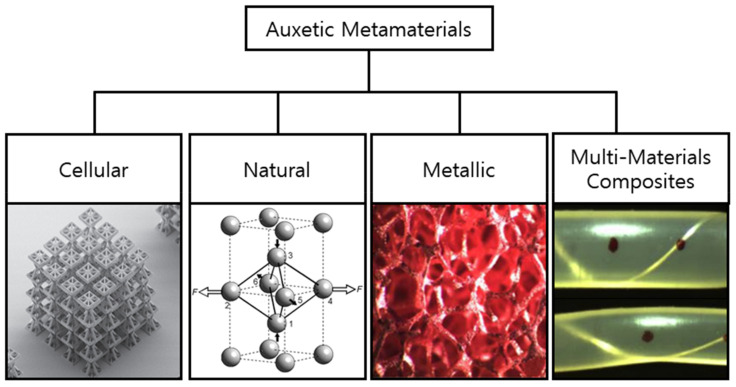
Classification of cellular auxetic metamaterials. Adapted from [21].

The term “auxetics”, derived from the Greek word “auxetikos” meaning “what tends to increase” was introduced by Evans in 1991 [14]. Auxetic structures or materials typically have a negative Poisson’s ratio. Materials with near-zero homogeneous Poisson’s ratios are known as “anepirretic materials” [15]. Other desirable material properties, such as superior shear resistance [16], indentation resistance [17], fracture resistance [18], synclastic behavior [19], and variable permeability [20], are also seen in auxetic materials. Figure 3 shows that based on their base material and internal structure, auxetic metamaterials can be broadly classified as cellular, natural, metallic, or multi-material composites [21]. 

The existence of natural auxetic materials was reported quite a while ago through classical elastic theory. Natural auxetic materials can be found in α-cristobalite, pyrolitic graphite, and elsewhere. Keskar et al. reported that α-quartz, the most common form of crystalline silica, also exhibits a negative Poisson’s ratio (NPR) under large uniaxial tension [22]. Dagdelen et al. observe that crystalline materials with the anisotropic mechanical behavior exhibit orientation-dependent directional Poisson’s ratios [15]. However, it is important that the existence of auxetic directions alone is not enough to make a material’s bulk average Poisson’s ratio negative [15]. Materials and structures that are based on metal and display auxetic effects are termed metallic auxetic structures. Multi-material auxetics are composed of more than one single base material, while auxetic composites are composed of both auxetic and non-auxetic materials. However, nearly all of the currently known homogeneously auxetic materials are porous foams or purposefully designed hinged meta-materials with open, re-entrant structures [23]. 

In this work, drawing inspiration from the works previously carried out by Ren et al. [21] and Kolken et al. [24], we present an overview of cellular auxetic materials and structures. Differences in geometrical configuration have been used to classify cellular auxetic materials and structures into structural deformation models, perforated and crumpled sheets models, and other miscellaneous models with unusual geometry. Although there are a number of reviews on auxetic materials, a comprehensive review which focuses on the applications of these materials is lacking. Through this review, we hope to provide a concise summary of the various cellular auxetic structures while ensuring special focus on reviewing the state-of-the-art applications of auxetic materials and structures in eclectic domains, such as sensors and actuators [25,26,27], sports sciences [28,29], textiles [30], medicine [31,32,33,34,35,36] and defence [37,38,39,40].

## 2. Cellular Auxetic Materials and Structures

**Figure 4:**
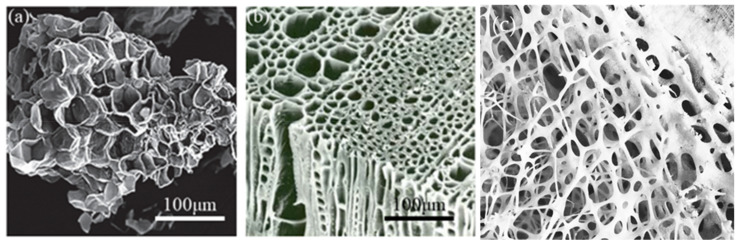
Examples of cellular microstructure in nature; (**a**) micro cork particle [44], (**b**) wood [45], and (**c**) bone [46].

Scheffler and Colombo maintain that “Cellular materials are formed by periodic or stochastic arrangements of open or closed cell types, with either two-dimensional cell configurations (such as honeycomb) or three-dimensional polyhedral layouts (such as lattice structures)” [41]. Cellular materials offer unique functional characteristics, such as high specific strength and stiffness, enhanced absorption of mechanical energy, and heat-transfer control, allowing design freedom beyond the capabilities of solid materials [42]. To achieve the desired application requirements, the properties of the cellular materials can be tuned by manipulation of cell topology and size [43]. Figure 4 shows examples of cellular microstructure in nature.

**Figure 5:**
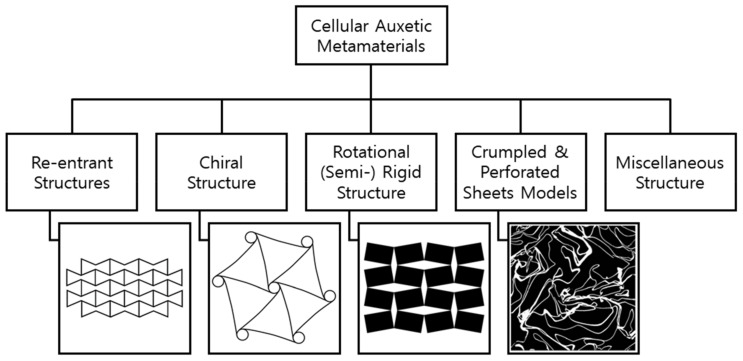
Classification of cellular auxetic metamaterials. Adapted from [21].

Figure 5 shows that cellular auxetic meta-materials can be categorized into re-entrant structures, chiral structures, rotational (semi-) rigid structures, crumpled and perforated sheet models, and other miscellaneous structures.

**Figure 6:**
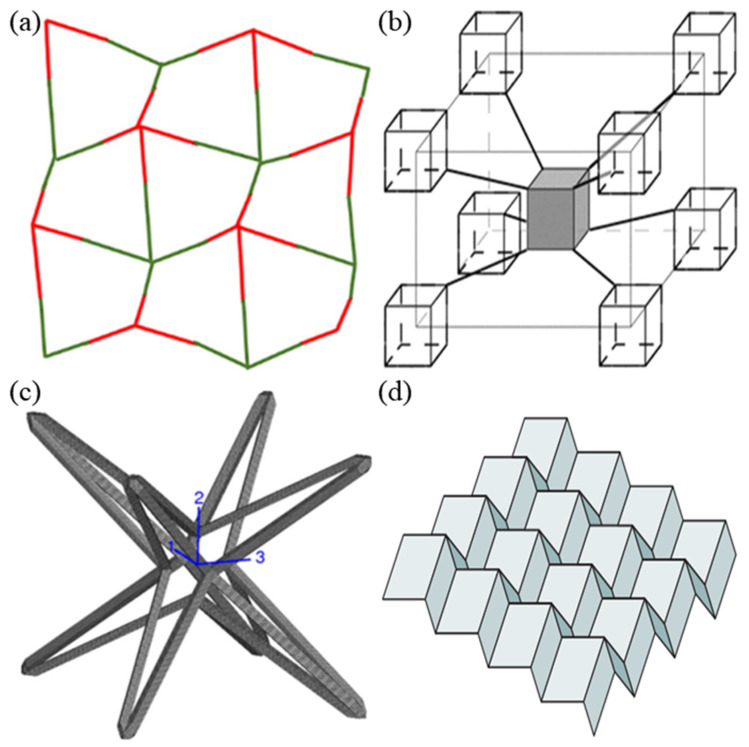
Examples of miscellaneous structure; (**a**) the egg rack model, (**b**) tethered nodule model, (**c**) hexatruss model, and (**d**) origami structure [21,47,48,49].

Miscellaneous structures include arbitrary geometries, such as the egg rack model, tethered nodule model, hexatruss model, and origami structures, as shown in Figure 6.

### 2.1. Re-Entrant Structures

**Figure 7:**
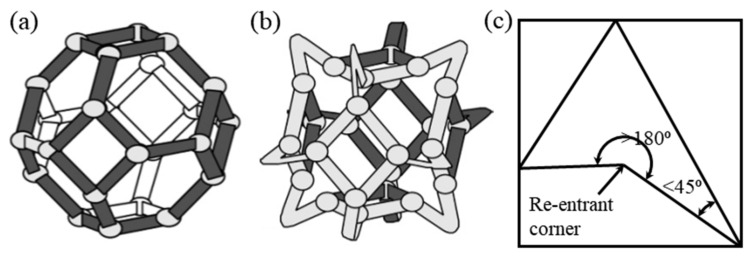
(**a**) Conventional cell, and (**b**) ideal auxetic cell [52], (**c**) re-entrant corner and re-entrant angle in irregular polygon [53].

Figure 7 shows both a conventional cell and an ideal auxetic cell. The re-entrant angle in an irregular polygon is an interior angle that is greater than 180°, as seen in the ribs of the “bow-tie” honeycomb [23]. Figure 7c shows the re-entrant corner and re-entrant angle in an irregular polygon. Re-entrant structures are formed by hexagonal face cells, which have the edges protruding outwardly. Along with re-alignment (hinging), deflection and axial deformation (stretching) of cell ribs are also responsible for the deformation of re-entrant structures and auxetic behavior [50,51].

**Figure 8:**
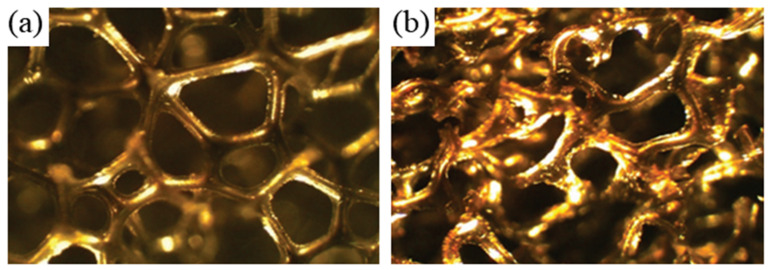
Micrograph of (**a**) conventional cylindrical foam, and (**b**) auxetic cylindrical foam [57].

Lakes proposed his foam transformation procedure that involved controlled heating and tri-axial compression to convert conventional, open-cell thermoplastic foams to foams that exhibit re-entrant structures in 1987, and began the development of intentionally designed auxetic materials [19]. After being tri-axially compressed in a mold, the foam was heated to a temperature above its softening temperature, and then cooled to room temperature, after which it could be extracted to undergo relaxation. Strict adherence to this procedure transforms conventional unit cells to re-entrant cells [19]. Figure 8a,b show conventional and auxetic foams, respectively. The type of foam obtained depends on the application conditions of tri-axial compression. Tri-axial compression during the forming process enables the transformation of thermosetting foams, whereas sequential tri-axial plastic compression is used to obtain re-entrant metallic foams [52,53,54]. Chan and Evans [55], Quadrini et al. [56], and Friis et al. [52] also proposed a number of transformation processes in terms of advantages (such as intact cell structure), and disadvantages (such as increased anisotropy).

**Figure 9:**
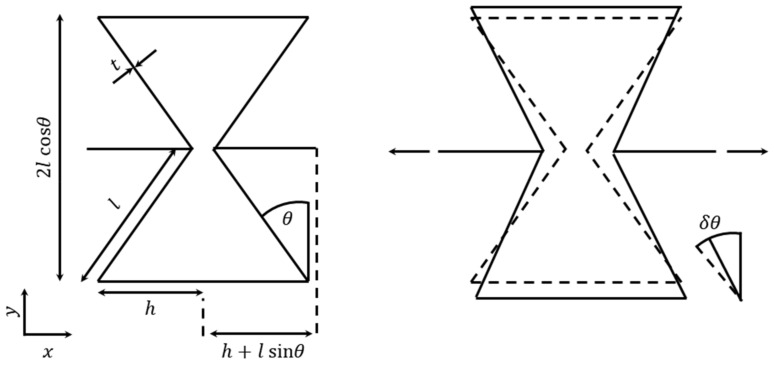
Hexagonal unit cell described by Masters and Evans [50].

The first traditional cellular structure in the form of re-entrant honeycombs was proposed by Gibson et al. [42] in 1982, and revealed that the auxetic behaviour is attributed to the flexure of diagonal ribs, as well as the outward movement of vertical ribs when the honeycomb is stretched horizontally. Masters et al. proposed a theoretical model for 2D re-entrant structures, which could predict the elastic constants of honeycombs based on the deformation of honeycomb cells by flexure, stretching, and hinging [50]. While regular hexagonal cells show in-plane isotropy, re-entrant hexagonal cells show high anisotropy [50]. To illustrate the behaviour of conventional and auxetic honeycombs and foams, Gibson et al. [42] provided a traditional 2D model. The Poisson’s ratio and Young’s modulus along the loading direction are given as follows:

(3) $$\nu_{\mathrm{yx}}=\frac{\sin\theta \;\left(h/l+\sin\theta \right)}{\cos^{2}\theta }$$

(4) $$E_{y}=k\frac{\left(h/l+\sin\theta \right)}{b\cos^{3}\theta }$$

(5) $$k=E_{s\;}b\;{\left(\frac{t}{l}\right)}^{3}$$

where, *h*, *l*, *θ* are defined as in Figure 9, *b* is the depth, and *E_s_* is the intrinsic Young’s modulus of the material forming the cell walls. 

**Figure 10:**
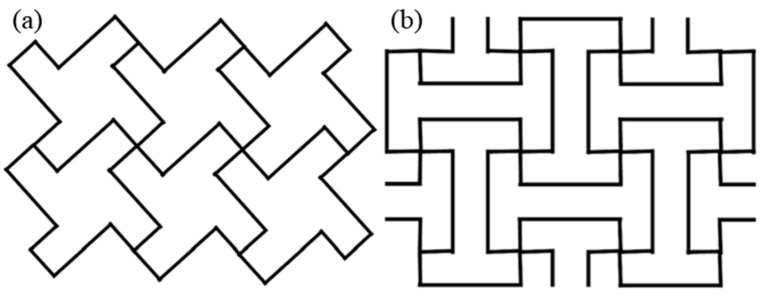
Auxetic foam geometries. (**a**) lozenge grid [24,63], and (**b**) square grid [24,63].

Other important auxetic foam geometries include the Lozenge (Figure 10a) and Square grids (Figure 10b), introduced in the missing-rib structure [51]. It was also shown that most of the differences between the conventional and auxetic foams are due to a change in cell geometry [58]. Lakes and Elms (2000), through the output of performing indentation tests, came to the conclusion that re-entrant foams exhibit higher yield strengths and energy absorption capabilities than the conventional foams of equivalent original density [59]. Several high-throughput methods are available, and include the micro-pillar compression [60], where the size of the micro pillar affects the measured properties; micro-tensile tests [61]; and nano-indentation techniques [62]. High-throughput methods can provide fine spatial resolution, rapid measurement speed, and have the possibility of being automated to determine mechanical properties, such as the yield strength of re-entrant foams.

**Figure 11:**
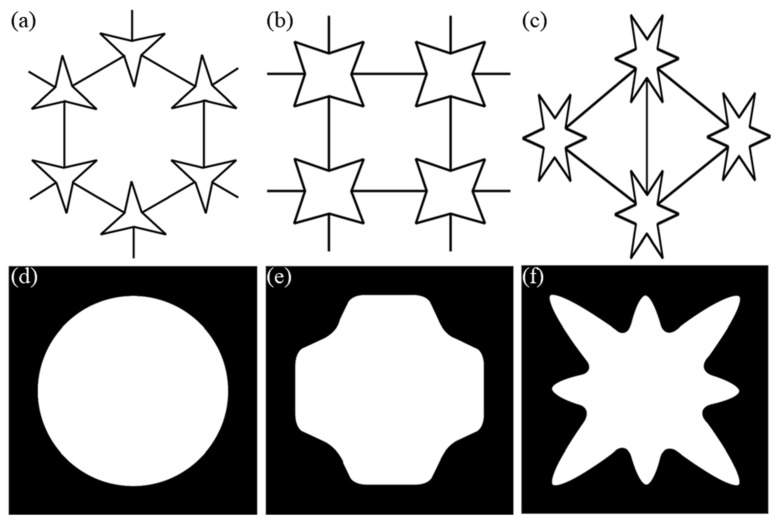
Re-entrant honeycomb structure: (**a**) 3-star, (**b**) 4-star, and (**c**) 6-star system [64]. Void shapes in 2D soft materials (**d**) exhibiting the highest stiffness, (**e**) exhibiting the greatest auxetic response, and (**f**) showing the least compaction with positive Poisson’s ratio [24,65].

Grima et al. in 2005 used the force field-based empirical modelling using dummy atoms (EMUDA) technique, which provides evidence that star-shaped systems have the potential for auxetic behavior. The STAR-3 system (Figure 11a) exhibits both auxetic and conventional behavior, while the STAR-4 and STAR-6 systems (Figure 11b,c) exhibit on-axis auxeticity for most combinations of force constants [64]. Investigations by Overvelde et al. [65] extended the concept of Poisson’s ratio to finite elasticity. They reported the effect of pore shape and porosity on structural response, and showed that B-Type voids (Figure 11e) initiated the highest auxetic response, whereas A-type voids (Figure 11d) showed the highest stiffness [65]. Figure 11 shows examples of star-shaped systems and systems with pre-designed voids or pores.

**Figure 12:**
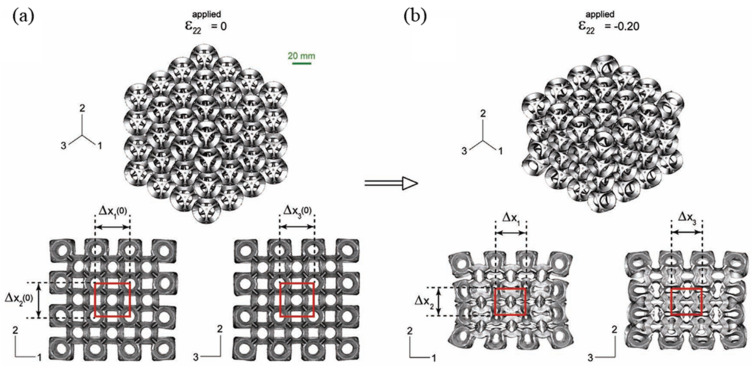
Experimental and numerical image of the 6-hole Bucklicrystal: (**a**) isometric and cross-sectional views of the undeformed crystal from micro-CT (micro computed tomography) X-ray imaging machine, and (**b**) isometric and cross-sectional views of the uniaxially compressed crystal from micro-CT volumetric data sets [71,72].

The first artificially built 3D auxetic system exhibiting *a ν* = −1, dates back to at least the structure proposed by Almgren [66]. Ever since, modifications in microstructure parameters such as strut length and re-entrant angle have produced a number of geometries exhibiting auxetic behavior. Koerner et al. [67] proposed an eigenmode analysis of a basic unit cell-based approach to systematically identify a variety of complex 2D and 3D auxetic structures. The auxetic behavior was ascribed to the rotation of nodal points or strut midpoints. They concluded that if these rotational points represented lattice symmetry, complete auxetic behavior would be obtained. Yang et al. [68] and Buckmann et al. [69] built several 3D re-entrant structures using additive manufacturing techniques. These works were important from the point of view of comparing the compressive strength, and it was shown that the compressive strength of highly auxetic structures could potentially be much higher than that of the regular, conventional foams [68]. An increase in the re-entrant angle increases the auxeticity of a unit-cell structure with re-entrant hollow skeletons [70]. The 3D extension of the buckling-induced pattern transformation in 2D, also known as “Bucklicrystals” in Figure 12, refers to the periodic arrangement of patterned spherical shells, which undergo isotropic volume reduction in response to a force stimulus, when all ligaments undergo a uniform first buckling mode [71,72]. Further investigations carried out by Shen et al. [73] and Lim et al. [74] proposed the elastomeric NPR structures based on simple initial geometries and the 3D anisotropic intersecting double arrowhead structure respectively. However, Yang et al. reported that for the models they studied, auxetic behavior not only depended on the re-entrant cell shapes, but also depended on the angle. 

**Figure 13:**
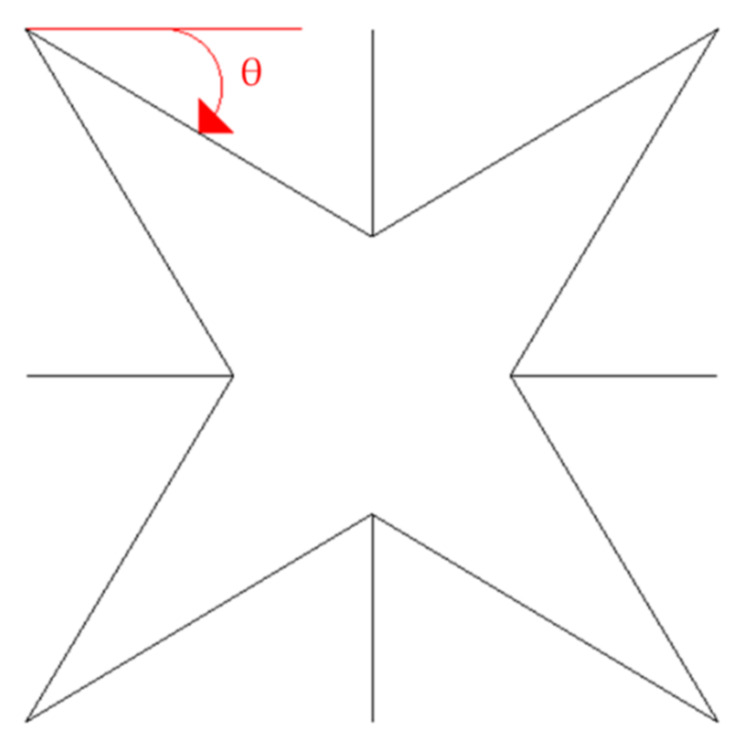
The auxetic angle *ϴ.* Adapted from [76].

Their models exhibited increasing auxetic behavior for auxetic angles (as shown in Figure 13) larger than 20° and lesser than 45° [68,75,76]. 

**Figure 14:**
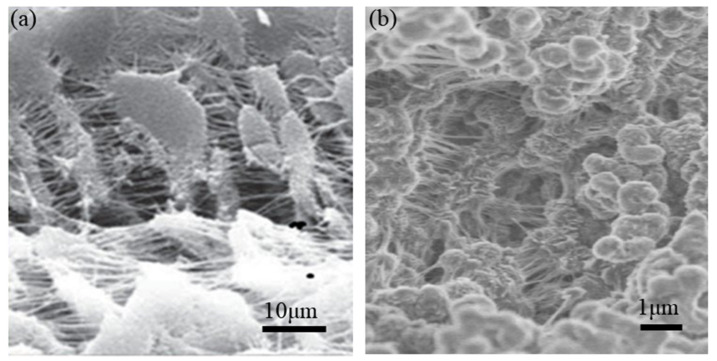
(**a**) Scanning electron microscope (SEM) image of the microstructure of polytetrafluroethylene (PTFE). (**b**) Low vacuum scanning electron microscopy (LVSEM) micrograph of GUR^®^ 1050 ultra-high molecular weight polyethylene (UHMWPE) powder showing fibrils and nodules of approximately 1 μm size [79,80,81].

Processed microporous polymeric structures of polytetrafluroethylene (PTFE) (Figure 14a) with disc-like structures and fibrils are highly anisotropic with negative Poisson’s ratio [77]. This auxeticity under an applied load is due to a cooperating network of interconnecting nodules and fibrils. A similar nodule-fibril structure can be achieved in ultra-high molecular weight polyethylene (UHMWPE) (Figure 14b) by the processes of compaction, sintering, and extrusion [78]. Expansion of the nodules in the transverse direction and the fibrils being pushed apart at the same time result in large negative Poisson’s ratio values.

### 2.2. Chiral Structures

**Figure 15:**
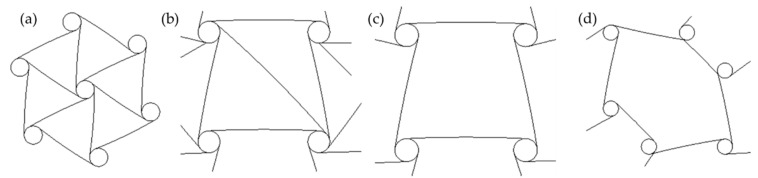
(**a**) 2D hexachiral and 2D meta-chiral systems with different number of ribs attached to each node (**b**) six ribs, (**c**) four ribs, and (**d**) three ribs [21].

**Figure 16:**
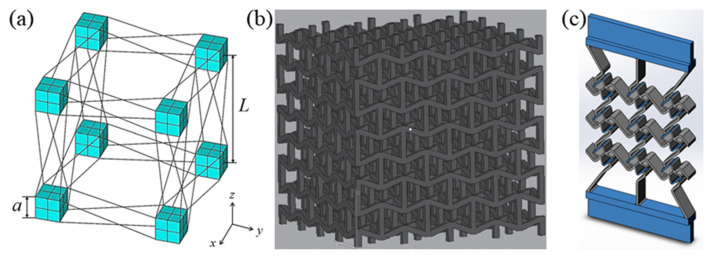
3D chiral lattice structure: (**a**) stretch-twist coupling lattice [21], (**b**) 3D re-entrant lattice [21], and (**c**) re-entrant core cell [83].

Even though symmetry is a scientific law of nature, asymmetry is also a main melody in our universe. From the basic components of living things, such as DNA and proteins, to the celestial bodies such as galaxies, asymmetry can be found everywhere. A chiral unit is comprised of a central cylinder encapsulated in tangentially attached ligaments, which is not superimposable on its mirror image, whereas anti-chiral structures exhibit reflexive symmetry [82]. Basically, a chiral structure is inseparable into two identical halves. Natural chiral materials include those found in flower petals and stems that climb in twisted fashion, including tendrils and twisted leaves. Chiral structures are classified into 2D chiral lattices, tri-, tetra-, and hexachiral honeycombs, meta-chiral structures, and 3D chiral lattices. Figure 15 and Figure 16a show examples of 2D and 3D chiral structures, respectively.

**Figure 17:**
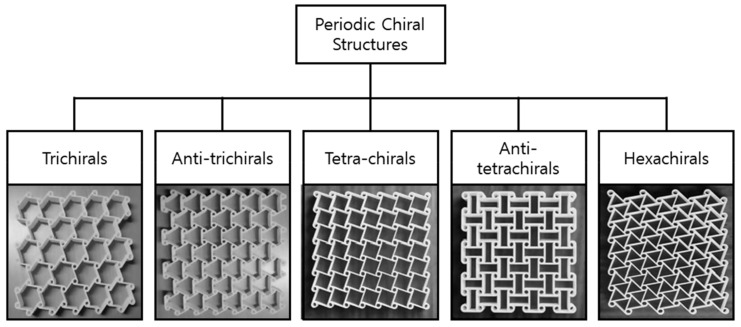
Classification and representative structures of periodic chiral structures [84].

The folding and unfolding of the ligaments under tensile and compressive loadings preceded by the rotation of the cylinders may result in a negative Poisson’s ratio close to −1 [84]. Periodic chiral structures can only be created by obeying the constraints of rotational symmetry i.e., for rotational symmetry of order *n*, the number of ligaments attached to each node should be *n*. Figure 17 shows that if these constraints are not relaxed, only five structures, trichirals, anti-trichirals, tetra-chirals, anti-tetrachirals, and hexachirals can exist [84,85].

Unlike the re-entrant cells, the Poisson’s ratio of chiral cells is not dependent on any structural angle [86]. However, it is interesting to note that the anti-tetra chiral honeycomb simultaneously displays anisotropy, auxeticity, and a lower shear modulus than usual cases [87]. The results in an investigation conducted by both analytical and finite elemental approaches on auxetic chiral models by Gatt et al. [88] indicated that the mechanical properties of the flexing anti-tetra chiral system were significantly impacted by the geometry and mechanical properties of the constituent materials. Specifically, the Poisson’s ratio was found to be dependent on the ratio of the ligament lengths and thickness.

A bridge between the chiral and anti-chiral structures, meta-chiral structures include the basic properties of both, and the Poisson’s ratio is dependent on the various aspect ratios and angles between the ligaments and nodes [85]. An infinite helical staircase is an example of a meta-chiral system. 

Three-dimensional chiral structures made of cubes and deformable ribs with regulatable Poisson’s ratio (−0.14) were extensively analyzed by Ha et al. [86] using finite element approaches. The lattices exhibited a stretch–twist coupling that increased with relative slenderness of ribs [86], and the Young’s modulus and effective shear modulus were found to be dependent on the number of unit cells per side. Jiang et al. [83] were able to integrate chiral and re-entrant structures by designing hybrid metamaterials, which were analyzed using mechanical experiments on 3D printed models and finite element simulations. These hybrid metamaterials had the re-entrant core cells in the center of a basic chiral cell. 

Auxetic 3D chiral lattices modelled and produced by additive manufacturing techniques, such as 3D direct laser writing [69], was made of Tango Black^®^ (Stratasys, Eden Prairie, MN, USA), a rubber-like additive manufacturing filament [89]. Some potential applications of the chiral models are proposed in the works of Airoldi et al. [90,91] and Budarapu et al. [92]. Airoldi et al. produced morphing wings for a variable camber wing-box using chiral honeycombs made of composite laminates. Budarapu et al. [92] proposed a framework to design an aircraft wing structure and analysed morphing air foils with chiral structure. Further studies could be carried out using an integrated approach comprising finite element modelling and additive manufacturing coupled with high-throughput methods, which could potentially accelerate the translation of chiral lattices into real-world products.

### 2.3. Crumpled and Perforated Sheets Models 

Crumpled sheet models are variations of planar sheet models, and their auxetic behavior is regarded as a consequence of a crumpled microstructure through the thickness [84]. With a few important assumptions, Zhang et al. [93] fabricated the crumpled sheets model, and concluded that the auxetic behavior could be increased when the crimped effect of the corrugated sheets is decreased. However, a decrease in crimped effect also decreases the stability of the structure during initial deformation. The assumptions included the following: that auxetic structure is perfectly periodic, straight ligaments of corrugated sheets are always kept straight under loading conditions, the shape of each corrugated sheet is formed by connecting straight ligaments with circular arcs, the thickness of corrugated sheets is ignorable, tubes are firmly connected with corrugated sheets, and no slippage takes place between pipes and corrugated sheets [93]. The previous studies on the auxeticity of single and double-layer carbon nanotube sheets (buckypaper) and graphene sheets have been carried out by Hall et al. [94], Scarpa et al. [95], Grima et al. [96], and Tan et al. [97].

**Figure 18:**
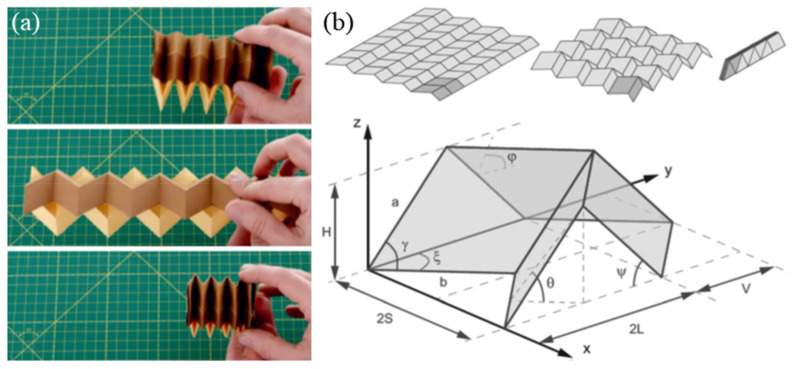
Origami metamaterials. (**a**) ‘zipper’-coupled tube system. (**b**) The geometrical design of Origami structure [100].

Crumpled sheet models are influenced by traditional paper-art techniques, such as ‘Kirigami’ and ‘Origami’, and have inspired several researchers to engage in studies of auxetic materials. Both Kirigami and Origami are Japanese paper arts, but unlike Origami, which only folds one piece of paper, Kirigami can cut paper as well. While the cellular fiber network structure in the sheet is responsible for the auxetic behavior of paper structures, the effect of materials and processing techniques on the auxetic behavior needs further research. Today, computer programs like Tree Maker [98] can be used to design various folding patterns of arbitrary complex three-dimensional structures, including Origami (Kirigami) auxetic structures [99,100]. The non-linear mechanical properties of Origami metamaterials are very interesting. A bi-stable behavior of unit cells is observed where switching of even one cell leads to a defect in the lattice [101]. Additionally, since the origami metamaterials can be extremely light yet rigid, they can be used for space applications as seen in the usage of Miura folding in deploying satellite solar panels in space [102]. Figure 18 is a typical example and design of an origami structure.

**Figure 19:**
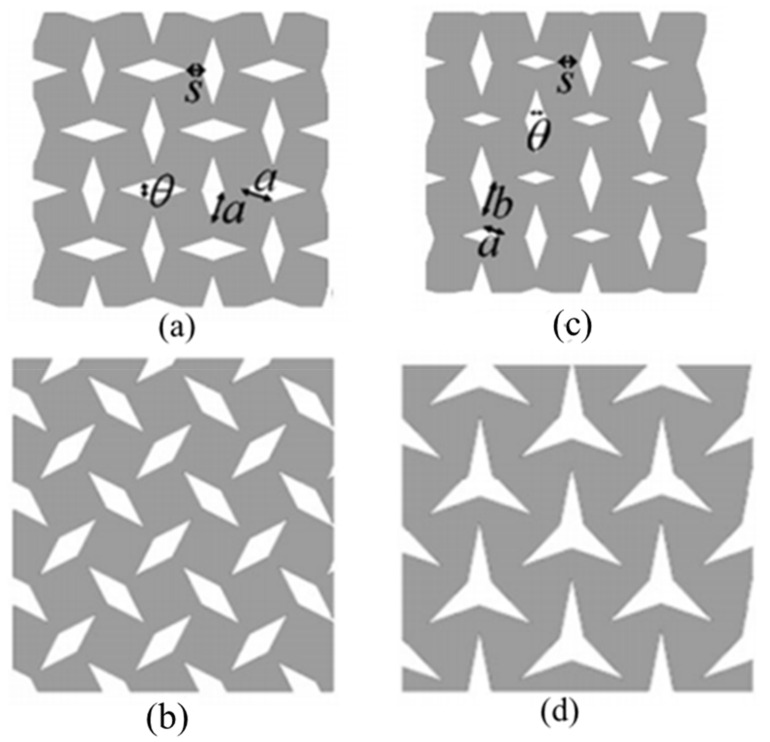
Perforated sheet systems. (**a**,**b**) Diamond-shape inclusions of the same size but at different orientations; (**c**) diamond-shaped inclusions of different sizes, and (**d**) star-shaped inclusions [21,103].

Extensive studies conducted by Grima et al. [103,104] on both star- and diamond-shaped cut perforated sheet models, as shown in Figure 19, and perforated sheet models with quasi random cuts, concluded that in spite of the arbitrariness of the cuts, the perforated sheets model still maintains its auxetic nature. The Poisson’s ratio function of perforated sheet systems can be represented as follows:

(6) $$\nu_{xy\;}=\frac{1}{\left(\nu_{yx}\right)}=\frac{a^{2}\cos^{2\;}\frac{\theta }{2\;}-b^{2\;}\sin^{2}\;\frac{\theta }{2}}{a^{2}\;\sin^{2}\;\frac{\theta }{2}-b^{2\;}\cos^{2\;}{\frac{\theta }{2}}^{}}$$

where, *a* and *b* are the sides of the rectangles, and *θ* represents the angle between the rectangles [21,103].

Further studies by Grima et al. [104] indicated that a high degree of symmetry is not necessary for the system to exhibit auxetic behavior. This study is pioneering, in the sense that it gives a tremendous freedom to the design of auxetic materials. It is a sort of flexibility, coupled with a materials development eco-system that will accelerate the development of new materials, and translate them into products.

### 2.4. Rotational (Semi-) Rigid Structures

A rotating (semi-) rigid system is another category of auxetic microstructures. The rigid squares connected by simple hinges can be viewed as an idealized rotating structure. When loaded, the squares will rotate at the vertices, either expanding or contracting depending on the loading type [105]. It is further categorized into rotating squares, rectangles, triangles, rhombi, and parallelograms. 

Assuming that the stiffness in the structure was due to the stiffness of the hinges, Grima et al. [105] used the conservation of energy principle to model and explore the auxetic potential of rotating rigid squares. Their studies showed that except for the semi-rigid rotating units whose Poisson’s ratios depended on the direction of loading and relative rigidity between these units and the hinges, the idealized system exhibited a constant Poisson’s ratio of −1. When these squares are replaced by rectangles, the system shows both positive and negative Poisson’s ratio, depending on the angle between the rectangles [106]. 

**Figure 20:**
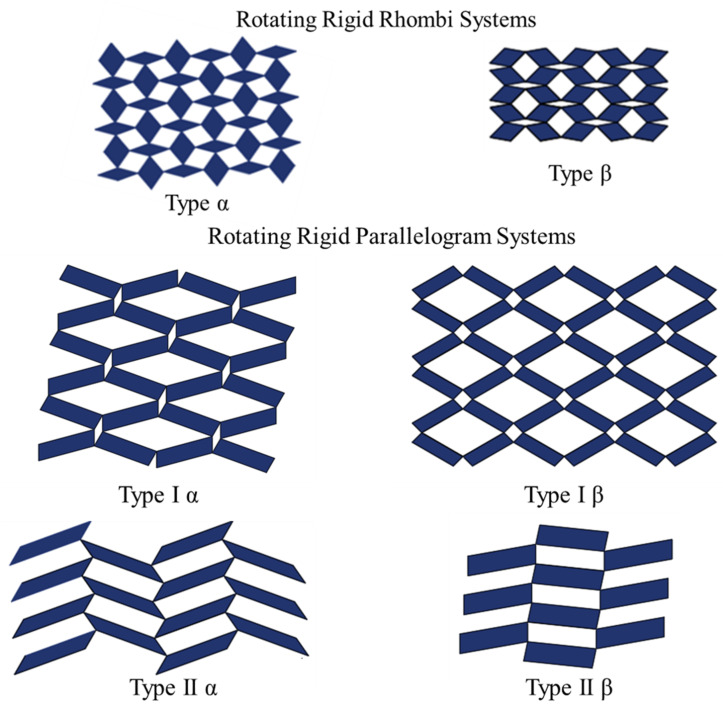
Rotating rhombi and rotating parallelogram systems; adapted from [108].

Grima et al. [105,106,107,108,109], Alderson et al. [110], and Rafsanjani et al. [111] have carried out extensive research on rotating polygon models. Their studies have shown that these systems depending on the configuration of the polygons and the openness of the system can exhibit both positive and negative Poisson’s ratios, and can be anisotropic [108]. Figure 20 shows rotating rhombi and rotating parallelogram systems. The rectangles can be classified into either Type I or Type II, depending on the shape they show in their empty space. If the empty space is rhombic shape, it is Type I, and if it is parallelogram shape, it is Type II. Rhombi are arranged into either Type α or Type β. If the obtuse angle of one rhombus is connected to the acute angle of its neighbor, it is a Type α system. If the connecting angles in both rhombi are the same, it is a Type β arrangement. According to Attard et al. (2008), both models can exhibit negative behavior [112]. The Type α system is highly anisotropic, while the Type β system shows in-plane isotropy. The Parallelogram scheme is a combination of the rectangle and rhombi schemes and has four categories: Type Iα, Type Iβ, Type IIα, and Type IIβ.

Grima et al. in 2006 [107] first provided a description of the mechanical properties of the rotating rigid triangles system. A hinged equilateral triangle system showed a Poisson’s ratio of −1, while isosceles triangle tessellates, depending on the shape of the triangles and the angle between them, exhibited both positive and negative Poisson’s ratios [107,113].

## 3. Applications of Auxetic Structures

As seen above, the auxetic materials exhibit counter-intuitive behavior. Due to their unique structural features, they have been used in many applications. When designing the auxetic meta-materials, the most desirable qualities are high stiffness and negative Poisson’s ratio, and several other aspects play a role in deciding the type of structure. Re-entrant structures offer a good balance between structural rigidity and negative Poisson’s ratio, although anisotropy may prove to be a challenge in certain applications (Kolken et al., 2017) [24]. Among the chiral structures, trichiral structures are potentially the least auxetic, whereas the anti-tetrachiral structures, due to their anisotropic nature, have good potential to exhibit high negative Poisson’s ratio. While re-entrant structures have been studied extensively, rotating rigid and chiral structures need more investigation. Properties that are generally most important from the product point of view are strength, fracture toughness, and energy absorption. Generally, auxetic materials are applied in fields where either one or a combination of the following properties is required [114]:

- Poisson’s ratio being negative or zero.
- Large shear resistance.
- Hardness improvement.
- Lower fatigue crack propagation.
- Large toughness and modulus resilience.
- Vibration absorption.

**Figure 21:**
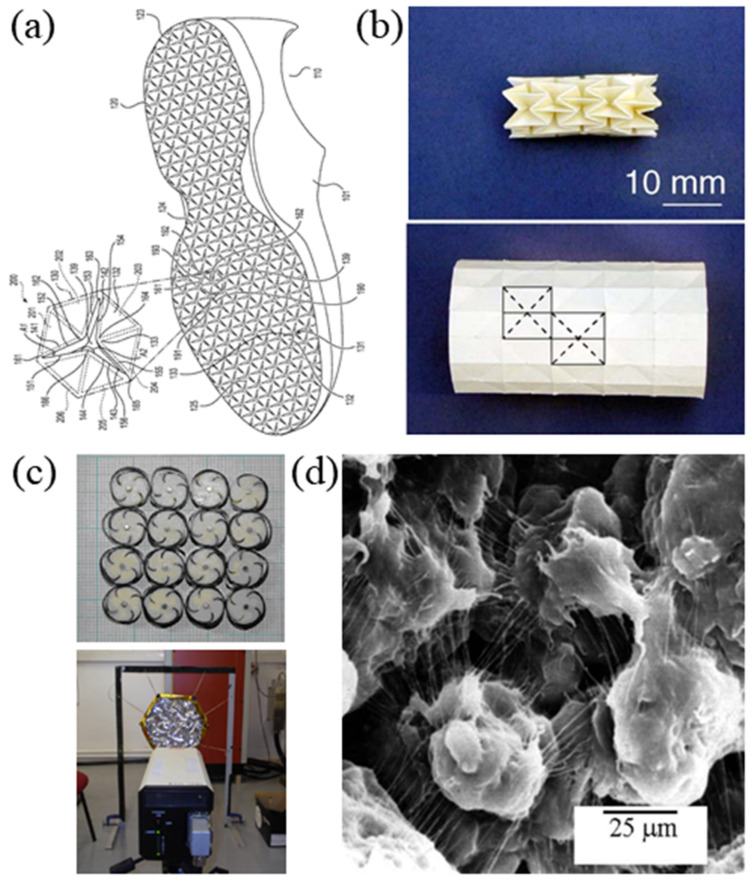
Applications of auxetic structure: (**a**) footwear (sports science) [115], (**b**) angioplasty stents (medical science) [116], (**c**) shape memory alloy cellular antenna (sensors and actuators) [117], and (**d**) auxetic polyethylene (textiles) [118].

Figure 21 shows photograph of the four examples below: sports science, medical science, sensors and actuators, and textiles, respectively.

### 3.1. Sports Science

Traditionally, the sports industry has been at the forefront of embracing new technologies and implementing them into products. As sports equipment improves, athletes can perform better, and the risk of injury decreases. Thus, it comes as no surprise that the first commercial products based on auxetic structures (foams and honeycombs) and materials are two commercial ranges of sports shoes. The sports equipment applied auxetic structures provide comfortability, safety, and durability better than common products. With fit and comfort being of the utmost importance for athletes, the Under Armour Architech sports shoe range [119,120] claims to aid conformability around domed shapes, and incorporates either an AM or molded auxetic re-entrant latticed upper. The Nike Free RN Flyknit sports shoe [115,121] is claimed to exhibit a bi-axial growth for improved traction and energy absorption, and employs an architectural closed cell foam outsole with an auxetic rotating triangles structure. A better conformability for support and improved energy absorption for thinner and lighter components make auxetic materials tremendously useful for application in sports protective devices, such as pads, gloves, and helmets. The Trust Helmet Pad System [122], marketed by D3O, incorporates pads with a re-entrant auxetic geometry, which are claimed to provide better fit to the head, and decreased acceleration under blunt impact. With further development, the auxetic materials could be used to manufacture low-weight bicycle frames, body-conforming swimsuits, etc.

### 3.2. Medical Industry

In the medical industry, various and suitable medical appliances are always needed for surgery. One of the famous examples of the medical device is the stent made of shape-memory alloy. Applying an auxetic structure to these devices provides stability and high-performance. Auxetic foam and honeycomb filters help clean the fouled filters, adjust pore size and shape, and compensate for the effects of pressure build-up due to fouling better than non-auxetic filters, since stretching the auxetic filters improves the performance by opening pores in both directions [31]. Auxetics are widely utilized in the manufacture of angioplasty stents [116], annuloplasty rings [32], and oesophageal stents [33,34], where they are used as dilators. Hamzehei et al. [123] developed anti-trichiral stents with equilateral triangular cores. The triangular cored stents exhibited up to three times better energy absorption capability than conventional anti-trichiral stents. Geng et al. using the selective laser sintering (SLS) technique of additive manufacturing, manufactured and analyzed cylindrical stents with chiral microstructures [35]. The stents exhibited auxetic behavior and their NPR depended on the circumferential strut number and the angle between the axial ligament and axial direction. Recently, Lin et al. used 4D printing and genetic algorithms to develop personalized shape memory polymer vascular stents exhibiting auxetic nature [124]. 

Auxetic materials have been used in the development of prosthesis liners as they can guarantee a secure hold on the prosthetic socket by reacting to the increase in distance due to a shortening of the amputation stump [125]. Paxton et al. designed and used the melt electro-wiring technique to fabricate auxetic tubular scaffolds [126]. Vijayavenkataraman et al. incorporated the re-entrant honeycomb and missing rib structures in the design of orthopedic bone plates and fabricated them using direct laser metal sintering technique [127]. In terms of stress-shielding and intra-operative bending, the re-entrant honeycomb incorporated bone plates were found to be better than their conventional counterparts. The potential use of microporous hollow auxetic structures as mechanical lungs is also under investigation. Auxetic structures are also used in medical bandages. Due to NPR, the swelling of the wound pushing on the bandage results in the controlled release of the wound-healing agent [36]. Recently, Yao et al. designed and fabricated auxetic bone screws using the selective laser melting (SLM) 3D printing method [128]. It was found that auxetic structures could improve the bone screw fixation. 

### 3.3. Sensors and Actuators

Auxetic mechanical metamaterials with their negative Poisson’s ratio are used in stretchable strain sensors, and provide great sensitivity (24-fold improvement over conventional sensors) [26]. This piezoresistive sensor designed by Jiang et al. had a re-entrant auxetic structure and it could stretch up to 98%. The practical application of this stretchable sensor was demonstrated by detecting the radial artery pulse from a healthy human female. Wong et al. 3D printed an auxetic strain sensor made of ionogel and found that the auxetic sensor had a better operational range as compared to analogous conventional films. Relative to the continuous film, the ionogel sensor with auxetic geometry exhibited up to 310% more extension [129]. Using a melt electro spinning technique the auxetic microfiber sheets (AMSs), auxetic solid sheets (ASSs), microfiber sheets (MSs), and solid sheets (SSs) have been fabricated for the design of stretchable force sensors for use in hand rehabilitation [27]. Using a femtosecond laser device to make the auxetic structures, gold as the sensing particle, poly (ε-caprolactone) (PCL) as the substrate and the melt electro-spinning technique Ko et al. designed a stretchable force sensor [27]. De Bellis and Bacigalupo studied the mechanical and piezoelectric response of anti-tetrachiral lattice structures with a view to designing piezoelectric sensors [130]. Li et al. designed stretchable force sensors by assembling carbon nano-tubes onto specific substrates. A tunable Poisson’s ratio was an important property of the substrates. For *a ν* = −0.5, the auxetic force sensor exhibited piezoresistive ability that was 300% better than conventional sensors [131]. Recently, using an auxetic structure made up of silicon rubber and chopped carbon fiber and manufactured using a 3D printing technique, Taherkhani et al. fabricated an auxetic sensor which is highly sensitive in low strain [132]. The sensitivity to low strain makes this sensor potentially useful in applications such as measurement of vibrations of the earth and wrist pulse. Using hierarchical auxetic structures and Ag composites, Han et al. developed bi-axially stable, high-performance conductor [133]. Auxetics have also been incorporated into hygroscopic sensor. Owing to the negative Poisson’s ratio, the sensor matches perfectly the deformation of the skin in the ankle, is durable, and provides stable performance in varied environments [25]. Farhangdoust et al. have recently proposed a micro electro-mechanical system (MEMS) with an auxetic design for a flexible membrane. For a harmonic pressure input compared to the conventional plain membrane, the auxetic membrane demonstrated excellent output sensitivity [134]. Currently, efforts are being made to produce the next generation of sensors by integrating auxetic materials and biomimetic principles.

Shape-memory properties coupled with auxetic structures have been used to manufacture antennas [117], and some deployable structures that needed no external actuation [135]. A star-shaped re-entrant lattice structure made up of four bi-metallic strips was proposed by Ai et al. [136]. The proposed structure had a tunable Poisson’s ratio and could be used in antennas and precision instruments. Sedal et al. used the Kirigami structures for development of deployable crawling robot [137]. Using preconceived auxetic structures to induce mechanical instabilities, Park et al. developed a new ‘instability-induced morphable structures’ called “Active skins” [138] which could be used for rapid surface deployment. Auxetic materials can also be used in the design and manufacture of hydrophones, because their low bulk modulus makes them more sensitive to pressure changes [139]. An interesting application is the auxetic porous membranes grafted by polymers, which could be used in valves and sensors [140]. Grima et al. incorporated magnetic components into several common auxetic structures and used external magnetic-fields to tune their macroscopic properties [141]. Left- and right-handed cylinder-based linear actuators have been designed using the handed shearing auxetic structures [142]. Depending upon the configuration, these handed structures can be used to create 2-Degree-of-freedom and 4-degree-of-freedom actuators [143]. A soft cylindrical actuator which could exhibit reversible flexural and twisting motion was developed by Lazarus et al. [144]. These actuators were built by taking advantage of the void-patterned cylindrical shells that exhibited auxetic behavior. Hasse et al. made use of an auxetic re-entrant structure and proposed a tube-like bending actuator where a difference in pressure between the internal and external surfaces of the tube could generate circumferential actuation [145]. A pneumatic 3D printed needle driver which facilitates linear actuation in surgical instruments was proposed by Pfeil et al. [146]. The driver used a re-entrant auxetic structure and was able to move and guide a needle due to the combined effects of pneumatics, inchworm kinematics and multimaterial additive manufacturing. To accelerate and streamline the development of metamaterial actuators, Bonfanti et al. proposed a computational method which leverages a combination of reinforced Monte Carlo method and discrete element simulations. Going one step further, they also showed that it was possible to design mechanical actuators by training a deep neural network [147]. 

Auxetic structures are increasingly being used in the development of energy-harvesting systems. Zhang et al. fabricated the first contact-mode triboelectric nanogenerator [148]. The self-powered strain sensor, built to sense body movements, was made out of auxetic polyurethane foam with a buckled structure, conductive fabric and PTFE. Auxetic cantilever beam energy harvesters (ACBEH) have been developed recently to enhance energy harvesting from ambient vibration. Simulation results indicated that the ACBEH could produce up to 2.51 times the power generated by plain cantilever energy beam harvesters [149]. Auxetic structures are also being used in intermediate boosters to enhance the energy absorption capacity of conventional cantilever beam energy harvesters [150]. 

### 3.4. Textiles

In order to apply auxetic materials to textile products, it is necessary to provide properties, such as comfortability, high energy absorption, high volume change, and wear resistance. There are two ways to manufacture auxetic textile materials. One is to use auxetic-based fiber directly, while the other is auxetic structure textiles made from conventional fiber. The first auxetic textiles were produced in the form of polypropylene (PP) fibres by Alderson et al. [118], using a partial melt extrusion process. The auxetic PP fibres have been produced using novel modifications to a conventional polymer processing technique, and as a result, their Poisson’s ratio is negative. An important application of the auxetic textile materials are the auxetic blast curtains [151] that are made from a helical auxetic yarn. These curtains achieve their blast-proof function by opening up when the pressure wave comes, and in the process, capturing the glass pieces and shrapnel. The synclastic nature of auxetic materials can be utilized in the manufacture of protective equipment for the elbow and knee joints, which are especially necessary for adventure sports. Similar to the auxetic blast curtains, bullet proof vests can be made out of auxetic materials, which upon impact will become thicker, rather than becoming thinner [152]. These types of auxetic fibre are available commercially, including Gore-Tex and PTFE [153].

### 3.5. Defence

The first reported instance of real-world application of negative Poisson’s ratio materials in defence dates back to at least the work of Garber [37]. Garber made use of NPR materials in aerospace, for enhanced thermal protection using pyrolitic graphite. Ma et al. developed a functionally graded NPR material concept for blast protection. The microstructure of the metamaterial was designed such that the stiffer ones were in the central region and the overall microstructure varied along the in-plane direction [154]. Auxetic structures such as the honeycomb [38] and chiral structures [39] have been subjected to blast response studies. Due to their ability to adapt to dynamic loading, compared to their conventional counterparts, auxetic materials exhibited better impact resistance by being able to draw materials into the impacted zones. Yang et al. [155] and Qi et al. [156] studied re-entrant auxetic cored sandwich panels. It was found that the in-plane perforation resistance of the auxetic panels in ballistic resistance was better than rectangular, hexagonal honeycomb and aluminium foam-cored panels of similar dimensional parameters and mass. Madke et al. investigated the high-velocity impact response of semi-auxetic braided composite face sheets with two different 3D re-entrant lattice cores [157]. They also conducted an interesting case-study on a body armour plate made of ultra-high molecular weight polyethylene (UHMWPE) reinforced with auxetic slattices. Luo et al. proposed and designed an auxetic re-entrant blast wall (ARBW) to improve anti-explosion performance of blast walls in off-shore platforms [158]. Since curved structures can support external loads better owing to their curvature, Lan et al. designed a novel cylindrical double arrowhead cored sandwich panel [159]. The results of extensive investigations on sandwich structures show promise in bringing about a manifold improvement in the quality of blast curtains, bullet-proof vests and bullet-proof helmets The worldwide market for these protective products is huge.

Another interesting application of NPR structures is the development of ultralight wheels and runflat tires [40]. The performance of the run-flat tires is potentially equivalent to that of current military vehicle tires but at almost half the weight. The tires may also be fully compatible with central tire inflation systems (CTIS). The emergence of organizations such as Auxetix Ltd. [160] which specially focus on blast and ballistic protection technologies will be helpful in quicker translation of these military-related applications into mass products. Re-entrant quadrangular lattices have been incorporated into the design of composite structures for smart airfoils [161]. Recently, Zhao et al. conducted uniaxial compression tests on thermally treated Beishan granite and found that for maximum thermal treatment temperature of 650 °C, Poisson’s ratio as low as –0.18 could be obtained [162]. While there needs to be further research to understand the principal mechanism responsible for the auxetic behaviour in thermally treated Beishan granite, it could potentially be used as a natural rampart in military fortifications.

Apart from these, the auxetic materials could find applications in several other vital areas, such as agriculture, for the controlled delivery of seeds and fertilizers; paints and dyes, where auxetic material-based paints could completely eliminate the formation of scratches; and in combination with other materials they could also be used for waterproofing.

## 4. Conclusions

### 4.1. Remarks

Moving away from a trial and error method to a scalable, collaborative approach that leverages the talent and efforts of the entire materials community is key to the design of manufacturable tailored microstructures [163]. This would facilitate the accelerated development of designer materials, and better cater to the needs of an ever-increasing population and dwindling resources. The use of repositories, such as the Materials Project [163], and tools, such as Pymatgen [164] and PyMKS (Materials Knowledge Systems in Python) [165], in conjunction with first-principles density functional theory (DFT) calculations, should be explored. They offer potentially effective ways of combining materials databases and intelligent materials design to accelerate materials discovery. Trailblazing studies carried out by Dagdelen et al. [15] reported the prediction of three previously unidentified homogeneously auxetic materials, as well as a number of anepirretic materials. Of these, a high-temperature phase of aluminum orthophosphate: HT–AlPO_4_, exhibiting a structure similar to α-cristobalite, but possessing a lower crystal symmetry due to its stoichiometry, is analytically predicted to have a Poisson’s ratio of –0.28, but needs further investigations of its auxetic behavior [15,166] Developments in additive manufacturing and computing have led the research community to the brink of a paradigm shift in the materials development and design cycle.

### 4.2. Conclusions

This paper focuses on providing a broad and introductory overview of auxetic materials and structures with a particular focus on cellular auxetic materials, in order to positively contribute to speeding up the material development cycle. Due to the inherent physical properties of auxetic materials, such as the negative Poisson’s ratio, various devices can be developed that were impossible with ordinary materials and structures. 

Over the past three decades, ever since Lakes reported the first auxetic re-entrant foam structures in 1987, we have seen tremendous progress in the materials. However, many important limitations must be addressed, before the auxetic materials are converted into commodities for mass consumption. The challenges to be solved include the high costs and limitations of additive manufacturing techniques, the inevitable porosity in additively manufactured auxetic structures which negatively affects their mechanical capability under load and a lack of 3D hierarchical systems which demonstrate satisfactory secondary behavior in compression. These limitations can tremendously compromise mechanical performance. The active contribution of high-throughput technology development and the whole material development ecosystem can be a game changer in eliminating these limitations, accelerating this material development cycle, and translating new materials, like the auxetic materials and structures, into products.
